# Evaluating the safety and efficacy of recombinant human thrombopoietin among severe sepsis patients with thrombocytopenia: study protocol for a randomized controlled trial

**DOI:** 10.1186/s13063-015-0746-6

**Published:** 2015-05-19

**Authors:** Qin Wu, Jianan Ren, Gefei Wang, Guosheng Gu, Dong Hu, Song Liu, Gunawei Li, Jun Chen, Ranran Li, Zhiwu Hong, Huajian Ren, Xiuwen Wu, Yuan Li, Min Yao, Yunzhao Zhao, Jieshou Li

**Affiliations:** Department of General Surgery, Jinling Hospital, Medical School of Nanjing University, 305 East Zhong Shan Road, Nanjing, 210002 China; Department of Surgery, VA Medical Center, 830 Chalkstone Avenue, Providence, RI 02908 USA

**Keywords:** Sepsis, Thrombocytopenia

## Abstract

**Background:**

Sepsis is still a major health problem that causes high mortality in all populations. Organ dysfunction including sepsis-associated thrombocytopenia is prevalent among sepsis patients, resulting in increasing mortality rates. Considering the clinical role of platelets, thrombocytopenia in sepsis has led to a large spend in research activity and clinical trials in this area, yet there is no consensus upon which treatment should be administered. As a result, platelet transfusion is often indicated to resolve low platelet counts, leading to an increasing risk of the multiple risks transfusion brings, such as infectious or immune system complications. Given the role of thrombopoietin in stimulating proliferation and differentiation of megakaryocytes, our previous study investigated the potential benefits of recombinant human thrombopoietin in severe sepsis patients with thrombocytopenia. However, there are several limitations in the study, which may have led to bias in our conclusion. Thus, we are conducting this study in order to evaluate the safety and efficacy of recombinant human thrombopoietin in a large, varied population.

**Methods/Design:**

The study is designed as a randomized, open-label, placebo-controlled, multi-center study in tertiary academic centers for evaluating the safety and efficacy of recombinant human thrombopoietin over placebo. An established total of 708 patients with sepsis and thrombocytopenia will undergo prospective random assignment to recombinant human thrombopoietin or placebo (a 1:1 ratio). The primary endpoint is 7-day all-cause mortality and 28-day all-cause mortality.

**Discussion:**

To our knowledge, this is the first study to evaluate the safety and efficacy of recombinant human thrombopoietin among severe sepsis patients with thrombocytopenia in a varied population. With our study, the level of evidence for the treatment of these patients will be significantly raised.

**Trial registration:**

ClinicalTrials.gov: NCT02094248. Registration date: 23 March 2014.

**Electronic supplementary material:**

The online version of this article (doi:10.1186/s13063-015-0746-6) contains supplementary material, which is available to authorized users.

## Background

Defined as a systemic deleterious host response to infection, sepsis is still a major healthcare problem around the world [1]. It is characterized by the cardinal signs of inflammation, including vasodilation, increased microvascular permeability and cell dysfunctions [2].

Despite their small size and anucleate status, platelets have diverse roles in vascular biology during sepsis. Not only are platelets the cellular mediator of thrombosis, but platelets are also immune cells that initiate and accelerate many vascular inflammatory conditions [3]. Due to a response to a systemic bacterial infection, continued platelet activation in sepsis patients is prevalent, leading to sepsis-associated thrombocytopenia [4]. Researches have suggested that the declining platelet counts (PCs) are associated with mortalities among sepsis patients caused by various diseases, indicating the importance of recovering from thrombocytopenia during sepsis [5, 6].

To date, evidence-based treatment strategies for sepsis-associated thrombocytopenia have not yet reached consensus for clinical practice. The management of thrombocytopenia remains a challenge. In the guidelines of surviving sepsis, platelet transfusion is advised to be administered when PCs are below 10 × 10^9^/L in the absence of apparent bleeding or below 20 × 10^9^/L if the patient has a significant risk of bleeding [1]. Studies have reported that early recovery from thrombocytopenia helps to prevent coagulopathy and decreases the mortality rate [7, 8]. Taking all of the above into account, more aggressive approaches to reverse the pathophysiological process of thrombocytopenia in severe sepsis are indeed required.

Several growth factors are involved in the process of platelet generation. Among them, thrombopoietin, which has been identified for its ability to stimulate proliferation and differentiation of megakaryocytes, has been identified [9]. Recent study has demonstrated the role of thrombopoietin as an effective stimulus for promoting hematopoietic stem cells when administered before radiotherapy to minimize hematopoietic stem cell injury and mutagenesis in mice, indicating the varied potentials of this factor [10].

Our previous study demonstrated that administration of recombinant human thrombopoietin (rhTPO) with conventional medical therapies could significantly improve the PCs in severe sepsis patients with thrombocytopenia and effectively reduce the platelet transfusion possibility [11]. We also observed significantly different mortality rates between the intervention group and the control group. This previous study is a single-center non-randomized prospective study. There are several limitations in the study, which may have led to bias in our conclusion. Thus, we have designed a clinical study that will evaluate the safety and efficacy of rhTPO in a large, varied population.

## Methods

### Objectives

The objective of this study is to determine the safety profile and efficacy profile of rhTPO in a large, varied severe sepsis population.

### Study design

The study is designed as a randomized, open-label, placebo-controlled, multi-center study in 17 tertiary academic medical centers. The sites that are participating in this study are medical, surgical or emergency ICUs. Patient enrollment is expected to last up to 18 months. The end of the study is defined by the last follow-up of the last enrolled patient. The trial is registered at clinicaltrials.gov (NCT02094248).

Qualified patients will be randomized into either of the two groups: the intervention group and the control group. Both groups receive standard care and will receive appropriate medical support based on guidelines issued by the surviving sepsis campaign [1].

The intervention group will receive administration of rhTPO (TPIAO™, Shenyang Sunshine Pharmaceutical Company Limited (SUNSHINE), Shenyang, China). The rhTPO will be subcutaneously injected in the intervention group after randomization. The dose is 15,000 U per day. The subcutaneous injection will be terminated when PCs are increased by 50 × 10^9^/L for 2 consecutive days compared with PCs at baseline. The duration of rhTPO will be 7 days. Time from randomization to administration of rhTPO will be within 24 hours.

Patients in the control group will receive the equal amount of normal saline, which will be subcutaneously injected as a placebo. The normal amount of saline is 1 ml per day for the initial stage and the maintenance stage. The subcutaneous injection will be terminated after 7 days. Time from randomization to administration of placebo will also be within 24 h.

### Recruitment

A principle investigator, assisted by a well-trained study coordinator in each participating center, will identify potentially eligible patients based on the eligibility criteria. And they, in collaboration with the treating physician, will confirm patient eligibility for the trial. The study coordinator will then obtain written informed consent from the patient. Patients will be consecutively enrolled at each of the participating centers whenever a study coordinator is available for enrollment. Consent will be obtained from participating clinicians before or at the time of patient consent.

### Eligibility and exclusion criteria

The detailed eligibility and exclusion criteria are listed in Additional file 1.

### Eligible, non-randomized patients

Eligible, non-randomized patients will be those who meet all inclusion criteria but are not included in the trial because of refusal of consent or inability to obtain consent within 12 h of the patient meeting the eligibility criteria. For these patients, we will collect data of platelets at baseline and at the time of eligibility, severity of illness at the time of eligibility, and their vital status in the ICU and at hospital discharge. The collection of these data will enable comparisons with randomized patients to assess threats to the generalizability of the trial results.

### Screening

Given the dynamic nature of the inclusion criteria, research coordinators will screen patients in each study center for eligibility once a day, either the morning or afternoon. Research coordinators will discuss all patients who meet the inclusion criteria, without an exclusion criterion, with the attending intensivist to confirm the inclusion.

### Randomization

Patients will be randomized after informed consent is obtained and it is confirmed that all inclusion and no exclusion criteria are met, prior to the patient-clinician discussion of treatment options and patient risk. Allocation will be based on a 1:1 ratio between the intervention and control groups. The allocation sequence will be generated by utilizing the ResMan Research Manager central randomization clinical trial management public platform (http://www.medresman.org/). The randomization will be stratified by study site. On the day of inclusion, investigators will log information into the web database using user identifications and passwords to obtain the randomization numbers and allocated groups for the eligible patients. Both investigators and participants will be aware of the allocations.

### Blinding

No blinding will take place in this study. Eligible patients who have given consent will be randomized into either of the two groups.

### Study flowchart

The study flowchart is listed in Additional file 2.

## Outcome measurements

### Primary outcome

The primary outcome of this study is the 7-day all-cause mortality and 28-day all-cause mortality.

### Secondary outcomes

The secondary outcomes of this study will include the following:

1. The number of participants who survive thrombocytopenia at the seventh day after randomization.
2. The total amount of platelets transfused into participants during the observational period.
3. The time to recovery to a normal platelet level.
4. The occurrence of bleeding events.

In this study, the normal platelet level is defined as PCs not less than 100 × 10^9^/L. Time to recovery to a normal platelet level is calculated from the date of randomization to the date when PCs >>100 × 10^9^/L.

### Data management

A case report form (CRF) is used for each patient to collect the data. Assessments are made at the screening period, inclusion (D0) and on days 1 to 7. Assessments include sepsis-related organ failure assessment, monitoring (catheter, tubes and drains, mechanical ventilation procedures), laboratory tests (hematology, biochemistry, urinalysis, pregnancy test at inclusion only), and microbiology (blood cultures, mycology). Data on adverse events, compliance and concomitant treatments are collected at each visit. We will determine vital status among survivors at 28 days following randomization through chart review and direct contact with patients if necessary. Participants will be evaluated every day till the seventh day after randomization. Physicians and research nurses who are not involved in the patients’ care will assess the outcome.

All documents collected in this study will be stored safely in confidential conditions. On all study-specific documents, other than the signed consent, the participant will be referred to by the study participant number/code, not by name. Study documentation will be archived for a period of 5 years after the study.

### Adverse events

All adverse events will be recorded and closely monitored until resolution or stabilization or until it has been shown that the study treatment is not the cause of the event. The chief investigator will be informed immediately of any serious adverse events and will determine (in cooperation with the treating medical practitioners) the seriousness and causality of these events.

All treatment-related serious adverse events will be recorded and reported to the research Ethics Committee as part of the report. Unexpected serious adverse events will be reported to the research Ethics Committee within the relevant time frames. The chief investigator will be responsible for all adverse event reporting. All site staff will be appropriately trained in the procedures to follow and the forms to use during the study protocol prior to study initiation. Regular central monitoring for all studies and site monitoring, as determined by the trial-specific risk assessment, will be used to ensure that all adverse events are identified and acted on appropriately.

## Statistical analysis

### Sample size and statistical analysis

The all-cause mortality of sepsis varies in different patient populations. The mortality of 28-day ranges from 20.8% to 61% but no reliable 7-day mortality has been reported to our knowledge [12, 13]. Using the 28-day mortality in our center, we assumed that the 7-day mortality is 40%. Our sample size was calculated to detect a 10% difference in mortality at day 7 between the two groups with a two-tailed test, a significance level (alpha) of 5% and a power of 80%. We plan to include a total of 708 subjects.

### Type of analysis

We will utilize an intention-to-treat approach to examine differences in the mortality in the two groups in the primary analysis by the Pearson χ2 test. Safety parameters and secondary efficacy parameters will be analyzed using the Pearson χ2 test and logistic regression (if necessary). Cox proportional hazards models and the Kaplan-Meier method will also be applied. Adverse events will be tabulated according to randomized group assignment and the proportions will be compared using Fisher’s exact test.

## Ethics

### Declaration of Helsinki

The research team will ensure that this study is conducted in accordance with the principles of the Declaration of Helsinki [14].

### Ethics committee and regulatory approval

The trial will also be conducted in accordance with the national ethics review for biomedical research involving humans (Trial) [15]; and international ethical guidelines for biomedical research involving human subjects [16].

The protocol, informed consent form, participant information sheet, any further patient documents and any proposed advertising material were submitted to the Institutional Review Board (IRB) of the Ethics Committee of Jinling Hospital for written approval. The research team has obtained approval from the Ethics Committee of Jinling Hospital for all substantial amendments to the original approved documents (approval number 2014SY-079).

### Protocol amendments

Any modifications to the protocol that may impact on the conduct of the study, the potential benefit of the patient or may affect patient safety, including changes of study objectives, study design, patient population, sample sizes, study procedures, or significant administrative aspects will require a formal amendment to the protocol. Such amendments will be agreed upon by the sponsor, and approved by the Ethics Committee prior to implementation and notified to the health authorities in accordance with local regulations.

Administrative changes of the protocol are minor corrections and/or clarifications that have no effect on the way the study is to be conducted. These administrative changes will be agreed upon by the sponsor, and will be documented in a memorandum. The Ethics Committee may be notified of administrative changes.

### Trial oversight

The oversight of this trial comprises three parts.

1. The Steering Committee. The committee will meet monthly by teleconference to review and address operational issues.
2. The Coordinating Center. The Department of General Surgery, Jinling Hospital, Nanjing, China, will serve as the central trial coordination and data management center. This department will work closely with the Steering Committee to coordinate trial activities, and will be responsible for managing trial sites, obtaining regulatory approvals, safety reporting, maintaining and archiving trial documentation, sourcing and distributing trial materials, developing electronic CRFs, training trial staff, performing data validation and monitoring activities, performing statistical programming and analysis, and providing results of analyses.
3. The Data and Safety Monitoring Board. The three members of the IRB are comprised of experts in statistics, critical care, and clinical research. The board members will meet by teleconference every month while the trial is actively recruiting. The primary role of the board will be to monitor adverse events among trial participants. The board will assess whether these events are linked to participation in the trial or to any aspect of the study protocol.

## Dissemination policy

### Trial results

The investigators will be involved in reviewing drafts of the manuscripts, abstracts, press releases, and any other publications arising from the study. Authors will acknowledge that the study was funded by the National Natural Science Foundation of China (81270478). The key point of this study design is summarized in Additional file 3.

### Authorship

Authorship will be determined in accordance with the International Committee of Medical Journal Editors guidelines and other contributors will be acknowledged.

## Discussion

This study is designed as an open-label study and physiological serum is used as a placebo. There is a possibility that the positive effect of rhTPO just lies among the surgical patients. For medical septic patients (for example pneumonia-derived sepsis), the role of rhTPO might be different. Thus, a placebo group in which physiological serum is administered could provide baseline data for the whole population. Also, the placebo group could be used to conduct a patient stratification analysis at the end of the study. So this study is designed as an open-label study and physiological serum is used as a placebo. The detailed reviewer’s comments and the author’s reply can be found in Additional file 4.

To our knowledge, this is the first study to evaluate the safety and efficacy of rhTPO among severe sepsis patients with thrombocytopenia in a varied population. With our study, the level of evidence for the treatment of these patients will be significantly raised.

## Trial status

The study was conceived and designed in 2014. We are now recruiting participants.

## Additional files

Additional file 1:
**Flowchart of this study.** The study is divided into three stages. Patients would be enrolled during the initial stage. After randomization, patients would receive treatment during the treatment visit stage. In addition, the follow-up stage would last until 28 days after enrollment. Additional file 2:
**Eligibility and exclusion criteria of this study.**
Additional file 3:
**Study summary.** The key message of this study protocol is summarized in this table. Additional file 4:
**The reviewer’s comments and the author’s reply.** We explain why this study is designed as an open-label study and physiological serum is used as a placebo.

## Abbreviations

CRF: case report form
PCs: platelet counts
rhTPO: recombinant human thrombopoietin
