# Supplementary material for: Evaluating the safety and efficacy of recombinant human thrombopoietin among severe sepsis patients with thrombocytopenia: study protocol for a randomized controlled trial
Source: Trials. 2015 May 19;16:220. doi: 10.1186/s13063-015-0746-6 (PMC4488939; doi:10.1186/s13063-015-0746-6)
Supplement: Additional file 1: — Flowchart of this study. The study is divided into three stages. Patients would be enrolled during the initial stage. After randomization, patients would receive treatment during the treatment visit stage. In addition, the follow-up stage would last until 28 days after enrollment. [file 13063_2015_746_MOESM1_ESM.pdf]

Additional file 1 Eligibility and Exclusion criteria of this study.

**Eligibility criteria**
1. Patients whose PCs were under 50×10^9/L.
2. Patients meet the criteria for severe sepsis, which is a modification of those defined by the surviving sepsis campaign
2.1 patients who have at least two or more of the following conditions:
2.1.1 Body temperature >38 or <36 ℃;
2.1.2 Heart rate >90 beats/min;
2.1.3 Respiratory rate >20 breaths/min or $PaCO_2$ <4.26 kPa;
2.1.4 White blood cell count $>12\times10^9$/L or $<4\times10^9$/L.
2.2 patients who have a microbiological evidence of a focal infection and/or a positive blood culture.

**Exclusion criteria**
1. Patients who are under recent chemotherapy (since less than six months)
2. Patients who have a history of malignancy with the last two years
3. Patients who have a history of bone marrow stem cell disorders
4. Patients who are currently enrolled in, or has completed within the last 30 days, another investigational device or drug study
5. Patients who are pregnant or at breastfeeding
6. Patients who have any kind of disorder that compromises the ability of the patients to give written informed consent and does not have a legally acceptable representative within 12 hours of patient meeting eligibility criteria
7. Patients who are less than 18 years or more than 85 years of age
8. Patients who have histories of bone marrow, lung, liver, pancreas, or small-bowel transplantation
9. Patients who have acute pancreatitis with no established source of infection
10. Patients who are under ongoing systemic immunosuppressant agents therapy, other than corticosteroids at doses < 2 mg/kg/day of prednisolone or equivalent
