# Supplementary figures and images for: Evaluating the safety and efficacy of recombinant human thrombopoietin among severe sepsis patients with thrombocytopenia: study protocol for a randomized controlled trial
Source: Trials. 2015 May 19;16:220. doi: 10.1186/s13063-015-0746-6 (PMC4488939; doi:10.1186/s13063-015-0746-6)

**Additional file 2 Flow chart of this study.**

**Initial stage:**

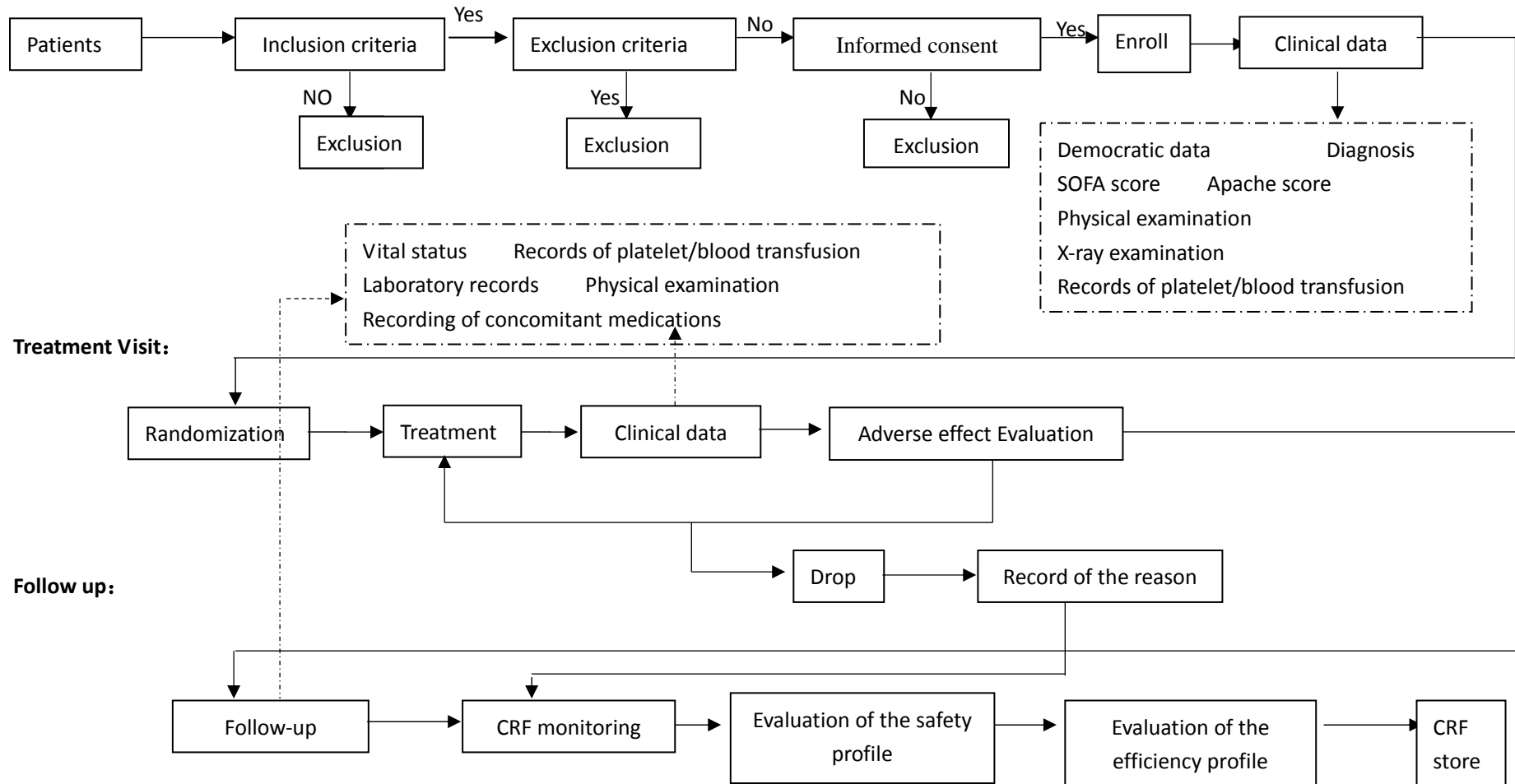

Supplement: Additional file 2: — Eligibility and exclusion criteria of this study. [file 13063_2015_746_MOESM2_ESM.pdf]
