# Supplementary material for: Evaluating the safety and efficacy of recombinant human thrombopoietin among severe sepsis patients with thrombocytopenia: study protocol for a randomized controlled trial
Source: Trials. 2015 May 19;16:220. doi: 10.1186/s13063-015-0746-6 (PMC4488939; doi:10.1186/s13063-015-0746-6)
Supplement: Additional file 3: — Study summary. The key message of this study protocol is summarized in this table. [file 13063_2015_746_MOESM3_ESM.pdf]

Additional file 3 Study Summary

Study Title	Evaluating the safety and efficacy of Recombinant Human Thrombopoietin among severe sepsis patients with thrombocytopenia: study protocol for a randomized controlled trial
Short Title	rhTPO in critical patients with thrombocytopenia
Protocol Number	NCT02094248
Methodology	A randomized, open-label, placebo-controlled, multi-center study
Study Duration	12 month
Study Sponsor	General surgery of Jinling Hospital, Nanjing, China
Objectives	The primary objectives of this study are to assess the safety and efficiency of rhTPO on mortality among critical patients with thrombocytopenia
Number of Subjects	703 patients
Main Inclusion Criteria	Sepsis patients with thrombocytopenia
Study Product, Dose, Route	rhTPO (Recombinant Human Thrombopoietin, TPIAO®, Shenyang Sunshine Pharmaceutical Company Limited [SUNSHINE], Shenyang, China), 15000 U per day, s.c injection
Reference therapy	Normal saline，1ml/day, s.c injection
Statistical Methodology	Intent to treatment
