# Supplementary material for: Evaluating the safety and efficacy of recombinant human thrombopoietin among severe sepsis patients with thrombocytopenia: study protocol for a randomized controlled trial
Source: Trials. 2015 May 19;16:220. doi: 10.1186/s13063-015-0746-6 (PMC4488939; doi:10.1186/s13063-015-0746-6)
Supplement: Additional file 4: — The reviewer’s comments and the author’s reply. We explain why this study is designed as an open-label study and physiological serum is used as a placebo. [file 13063_2015_746_MOESM4_ESM.pdf]

Additional file 4. The reviewer's comments and the author's reply

Comments: Platelets play a major role in coagulation, but also in innate immunity. This study aims to reduce early mortality (7 days) and at 28 days by stimulating platelet production using Recombinant Human Thrombopoietin in patients with sepsis and thrombopenia. The methodology is appropriate for the objectives, and the number of subjects to be included appears perfectly feasible in view of the number of participating centers. I have only one remark: Why do you plan to have a placebo using physiological serum, when the study is open-label?

Answers: thank you for your constructive suggestion. As you have mentioned, this study is designed as an open-label study and physiological serum is used as a placebo. Although our previous study has demonstrated that administration of recombinant human thrombopoietin with conventional medical therapies could significantly improve the platelet counts in severe sepsis patients with thrombocytopenia and effectively reduce the platelet transfusion possibility, this previous study still has some limitations. For the previous study, most cases of the sepsis are derived from trauma and surgical complications because this study was conducted in a surgical critical care unit. However, for this multi-center study, most of the participant centers are mixed critical care unit for both surgical and medical patients. There is a possibility that the positive effect of Recombinant Human Thrombopoietin just lies among the surgical patients. For medical septic patients (e.g. pneumonia-derived sepsis), the role of

Recombinant Human Thrombopoietin might be different. Thus, a placebo group which physiological serum is administrated could provide a baseline data for the whole population. Also, the placebo group could be used to conduct a patient stratification analysis at the end of the study. So this study is designed as an open-label study and physiological serum is used as a placebo.

Editorial requests:

Please include the reference number given with ethical approval with the ethics statement in the Methods section, and explain that ethical approval has been obtained.

Answers: We have added the statement that the ethics approval have been obtained.
